# Expression and Prognostic Value of ARID5A and its Correlation With Tumor-Infiltrating Immune Cells in Glioma

**DOI:** 10.3389/fonc.2021.638803

**Published:** 2021-05-19

**Authors:** Quan Zhou, Jinping Zhou, Jingyi Fan

**Affiliations:** Department of Pediatrics, Zhongnan Hospital of Wuhan University, Wuhan, China

**Keywords:** glioma, ARID5A, prognosis, immunity, CGGA, TCGA

## Abstract

AT-rich interaction domain 5A (ARID5A) is a member of the ARID family with a function that has been linked to autoimmune as well as inflammatory diseases. Some ARID family members are involved in the initiation and progression of human cancers. However, the function of ARID5A in glioma remains unknown. In this study, ARID5A expression levels were analyzed using the Gene Expression Profiling Interactive Analysis (GEPIA) database. Subsequently, the relationship between ARID5A expression and the clinical characteristics of glioma patients was evaluated using the Chinese Glioma Genome Atlas (CGGA) database and The Cancer Genome Atlas (TCGA) database. The prognostic value of ARID5A in glioma was estimated by Kaplan-Meier analysis and the receiver operating characteristic (ROC) curve analysis. Gene ontology (GO) analysis and gene set enrichment analysis (GSEA) were performed for functional prediction. The Tumor Immune Estimation Resource (TIMER) database was used to analyze the relationship between ARID5A and immune cell infiltration in glioma. Our results demonstrate that the expression of ARID5A was upregulated in glioma compared with that in nontumor brain tissues. High expression of ARID5A is associated with poor prognosis in glioma. We found that the expression of ARID5A was significantly upregulated with an increase in tumor malignancy. GO analysis revealed that co-expression genes of ARID5A are significantly involved in some important functions in glioma, and GSEA showed that multiple cancer-associated and immune-associated signaling pathways are enriched in the high ARID5A expression group. TIMER database indicated that ARID5A is correlated with tumor-infiltrating immune cells in glioma. Collectively, these findings indicate that ARID5A may be a potential prognostic biomarker and is correlated with immune infiltration in glioma.

## Introduction

Glioma is a common intracranial malignant primary tumor, accounting for about 27% of all central nervous system tumors (1). Glioma has high rates of morbidity, mortality, and recurrence, and a low cure rate (2, 3). Gliomas include low grade gliomas (LGGs) and glioblastoma multiform (GBM). LGGs, grade II and III gliomas, show slow growth and low malignancy; GBM is an invasive-growing tumor with high recurrence and mortality and is defined as grade IV glioma (4, 5). Due to their high degree of malignancy and invasive growth, gliomas are difficult to eradicate simply by surgery (2). At present, the primary treatment is maximum safe range of surgical resection, postoperative adjuvant radiotherapy, and temozolomide-based chemotherapy; however, the effect of these measures in prolonging the survival time of patients is still limited; the tumor recurrence rate is high, and patient prognosis is generally poor (6, 7). Despite this, the rapid development of gene sequencing technology has led to the discovery of many molecular markers that play an important role in the occurrence and development of gliomas. This development in technology is of significance for the diagnosis and prognosis of gliomas, and these biomarkers are expected to be used as molecular targets to improve tumor therapy in the clinical treatment of patients. In the 2016 World Health Organization (WHO) pathological classification of central nervous system tumors, isocitrate dehydrogenase (IDH) mutation and 1p/19q combined deletion were indicated as the two most critical molecular pathological biomarkers for glioma and have been widely used in clinical diagnosis and therapy (8). Therefore, it is of great significance to identify biomarkers for determining cancer risk, estimating prognosis of patients, and predicting tumor recurrence.

AT-rich interaction domain 5A (ARID5A) is a member of the ARID family, containing a helix-turn-helix ARID domain, which plays an important role in regulation of cell growth, differentiation, and development (9). Recently, many studies have indicated that ARID5A plays an important role in inflammation and immunity (10, 11). ARID5A can regulate the inflammatory response by stabilizing IL-6 mRNA (10). Furthermore, ARID5A can promote Th17 cell differentiation by stabilizing STAT3 mRNA to regulate the inflammatory response (11). ARID5A modulates the development of autoimmune and inflammatory diseases by stabilizing mRNA levels of several inflammatory molecules, suggesting that ARID5A may be a promising molecular target for the treatment of autoimmune and inflammatory diseases. Thus far, few studies have investigated the tumoral role of ARID5A; one study reported that ARID5A is related to the prognosis of lung cancer patients, where patients with high expression of ARID5A have a better prognosis (12). However, the expression pattern and biological role of ARID5A in glioma remains unknown.

In this study, to understand the potential role of ARID5A in glioma, we investigated the diagnostic and prognostic significance of ARID5A in glioma by data mining in the Chinese Glioma Genome Atlas (CGGA) and The Cancer Genome Atlas (TCGA) datasets. Subsequently, gene ontology (GO) analysis and gene set enrichment analysis (GSEA) were used to detect the possible biological functions and pathways of ARID5A in glioma. We then used the Tumor Immune Estimation Resource (TIMER) database to assess the relationship between ARID5A and tumor-infiltrating immune cells (TIICs) in glioma.

## Materials and Methods

### GEPIA Database Analysis

The online database GEPIA (Gene Expression Profiling Interactive Analysis, http://gepia.cancer-pku.cn/index.html) (13) contains RNA sequencing data of multiple tumors and normal samples from TCGA and the Genotype-Tissue Expression (GTEx) database, which can be used to analyze the differential expression of genes in tumors and normal tissues. We used GEPIA to analyze the difference of ARID5A expression between nontumor brain tissues and tumor brain tissues. Survival curve analysis was also performed using GEPIA, the effects of differential ARID5A expression on overall survival in LGG and GBM were analyzed.

### Data Collection and Bioinformatic Analysis

The CGGA database contains the bioinformatics data of more than 2,000 glioma samples from China, depicting the genomic and molecular genetic characteristics of glioma patients in China, and has guiding significance on the molecular typing and drug target development of glioma. We downloaded mRNA sequencing data and the corresponding clinical data of glioma patients from the CGGA (http://www.cgga.org.cn). After excluding data with missing clinical information, we obtained 807 glioma samples for further analysis, including 472 males and 335 females. According to the median expression level of ARID5A, the expression level of ARID5A in glioma samples was divided into the low-expression group and the high-expression group. Kaplan-Meier survival curve was used to analyze the influence of ARID5A expression on the survival of glioma patients. Correlation between ARID5A expression level and clinical data in glioma patients was detected; the accuracy of the prognosis model for glioma was then assessed using a receiver operating characteristic (ROC) curve.

TCGA is a public data platform containing 30 different cancer types and the clinical information of 11,000 patients. The research and mining of the TCGA database will contribute to the comprehensive understanding of the molecular mechanisms underlying cancer occurrence and development, and improve the ability of diagnosis and treatment of cancer. The LGG and GBM sequencing data and the corresponding clinical data of the samples were obtained from the TCGA database. Subsequently, we download GTEx normal brain tissue gene expression data from UCSC Xena (https://xena.ucsc.edu/). Based on this GTEx data, we analyzed the ARID5A expression level in different normal organs using the R language “ggpubr” package. The expression of ARID5A in 697 glioma samples and five nontumor brain tissues from TCGA and 1152 nontumor brain tissues form GTEx were analyzed. Correlation between the expression level of ARID5A and clinical data in glioma, Kaplan-Meier survival curve and ROC curve analysis in glioma were verified with TCGA data.

Pearson coefficient analysis was used to screen other genes related to ARID5A expression in the CGGA and TCGA databases. For the intersecting genes, the Database for Annotation, Visualization and Integrated Discovery (DAVID, https://david-d.ncifcrf.gov/) was used to perform GO analysis on 1858 genes positively related to ARID5A expression (Pearson *r* > 0.3, *P* < 0.01) and 260 genes negatively related to ARID5A expression (Pearson *r* < -0.3, *P* < 0.01).GSEA is an analysis method for genome-wide expression data. GSEA4.1.4 was used for analysis, and the expression level of ARID5A genes was divided into high expression and low expression groups. The gene set C2.CP. Kegg. V7.2.symbols was selected for single gene GSEA, and 1000 random combinations were used for enrichment analysis. The gene sets with *P* < 0.05 and FDR < 25% were taken as significantly enriched gene sets to analyze the possible role of ARID5A gene in gliomas.

### Immune Infiltration Analysis

The TIMER database (14) includes 32 tumors in TCGA and a total of 10,897 samples, which can comprehensively analyze TIICs. We used the TIMER “gene module” to analyze the correlation between ARID5A and glioma immune infiltration level, including the correlation with B cells, CD4+ T cells, CD8+ T cells, neutrophils, macrophages and dendritic cells. Kaplan-Meier survival curve was used to detect the influence of ARID5A expression and immune cell infiltration on patient overall survival. Univariate and multivariate Cox survival analysis was used to analyze the factors influencing the survival of patients.

### Statistical Analysis

The relationship between clinicopathological information and ARID5A expression in glioma patients was analyzed by the Wilcox or Kruskal test. The survival curve analysis was tested using the log-rank method. ROC curve analysis of the value of ARID5A expression in glioma diagnosis was also performed, and the relationship between ARID5A gene and immune infiltration level was analyzed using the TIMER database. *P* < 0.05 indicated that the difference was statistically significant.

## Results

### Expression and Survival Analysis by GEPIA

**Figure 1:**
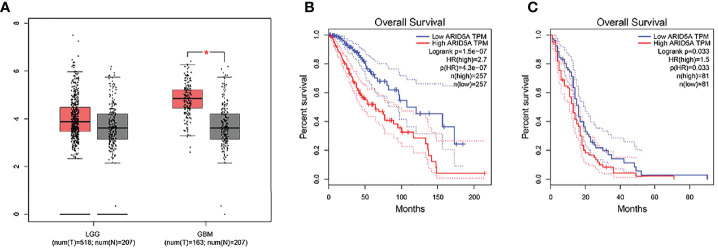
The expression of ARID5A in normal and glioma tissues (LGG+GBM) based on GEPIA **(A)**. Kaplan-Meier analysis of overall survival was performed in LGG **(B)** and GBM **(C)**.

Through GEPIA analysis of the LGG and GBM datasets in the TCGA database, we found that, compared with nontumor brain tissues, the expression of ARID5A was significantly upregulated in GBM tissues but was not significantly different in LGG tissues (**Figure 1A**). Using the GBM and LGG datasets from the GEPIA database for online survival analysis, we found that the expression of ARID5A was negatively correlated with the prognoses of LGG (**Figure 1B**) and GBM (**Figure 1C**), and the higher the expression level was, the worse the prognosis was.

### Low Expression of ARID5A in Human Brain Tissue

**Figure 2:**
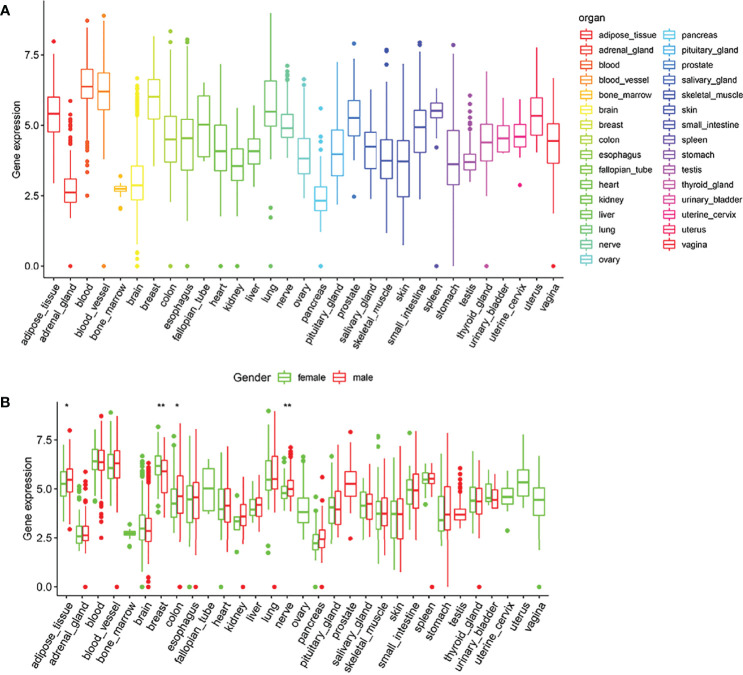
Expression box type of ARID5A in normal human tissues analyzed by GTEx data **(A)**. ARID5A expression level in tissues of different genders **(B)**. *P < 0.05; **P < 0.01.

According to the GTEx data, we explored the expression level of ARID5A in normal human tissues. We found that the expression of ARID5A was lower in the normal brain tissues than in most other tissues (**Figure 2A**), and there was no significant difference between men and women in normal brain tissues (**Figure 2B**).

### Relationship Between ARID5A Expression and Prognosis in Glioma Patients

**Figure 3:**
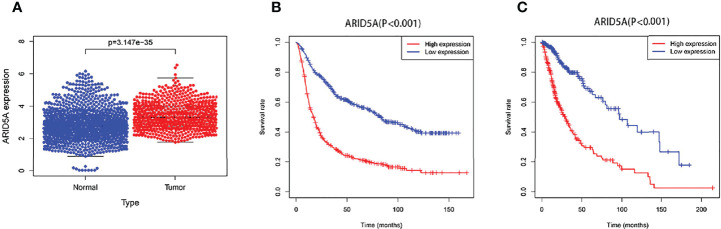
ARID5A expression in different normal human tissues and tumor tissues based on GTEx and TCGA databases **(A)**. Kaplan-Meier curves of overall survival in CGGA **(B)** and TCGA **(C)**.

We combined the RNA sequencing data from GTEx and TCGA databases to analyze the differences in ARID5A expression between normal and glioma tissues, including 1152 normal brain tissues from the GTEx database, five normal brain tissues and 697 glioma tissues from the TCGA database. We found that ARID5A expression in tumor tissues was significantly higher than that in normal tissues (*P <*0.001) (**Figure 3A**). Subsequently, CGGA and TCGA data were analyzed by Kaplan-Meier survival analysis, indicating that overall survival was lower in the high ARID5A expression group than in the low expression group (*P* < 0.001) (**Figures 3B, C**). The prognosis of patients with high ARID5A expression was significantly poorer than that of patients with low ARID5A expression, indicating that high expression of ARID5A is probably a marker of poor prognosis in glioma.

### The Relationship Between ARID5A Expression Levels and Clinical and Molecular Characteristics of Glioma Patients

**Figure 4:**
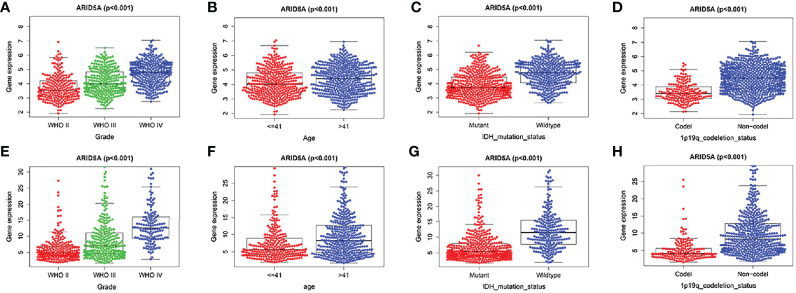
Associations of ARID5A expression with clinical and molecular characteristics in gliomas. Grade in CGGA **(A)** and TCGA **(E)**. Age in CGGA **(B)** and TCGA **(F)**. IDH mutant status in CGGA **(C)** and TCGA **(G)**. 1p19q codeletion in CGGA **(D)** and TCGA **(H)**.

We analyzed the correlation between ARID5A expression and WHO grade, age, IDH mutation,1p/19q co-deletion in glioma patients based on CGGA and TCGA RNA sequencing data and clinical and molecular characterization data. We found that the expression level of ARID5A increased with an increase in the WHO classification (*P* < 0.001) (**Figures 4A, E**). The expression level of ARID5A in patients aged > 41 years was significantly higher than that in patients aged ≦ 41 years (*P* < 0.001) (**Figures 4B, F**). Next, ARID5A expression was compared between IDH-wildtype and IDH-mutation gliomas. ARID5A expression was significantly higher in IDH-wildtype gliomas than in IDH mutant gliomas, and the difference was statistically significant (*P* < 0.001) (**Figures 4C, G**). Finally, we found that 1p/19q non-codeletion gliomas had a higher ARID5A expression level than 1p/19q co-deletion gliomas (*P* < 0.001) (**Figures 4D, H**). From the findings above, we can conclude that the increased expression of ARID5A was related to an increase in tumor malignancy.

**Figure 5:**
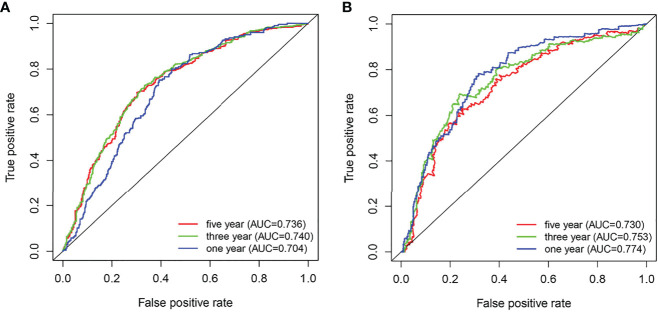
ROC analyses revealed the predictive value of ARID5A in glioma based on CGGA **(A)** and TCGA **(B)**.

Additionally, ROC curves were used to analyze the diagnostic value of the ARID5A gene in gliomas, and our results showed that the area under the curve (AUC) of ARID5A were 0.704, 0.740, and 0.736 for the CGGA datasets (**Figure 5A**) and 0.774, 0.753, and 0.730 for the TCGA datasets (**Figure 5B**) in 1, 3, and 5 years, respectively, indicating that the expression level of ARID5A had good diagnostic value for gliomas.

### Co-Expression Analysis and Gene Ontology Enrichment Analysis

**Figure 6:**
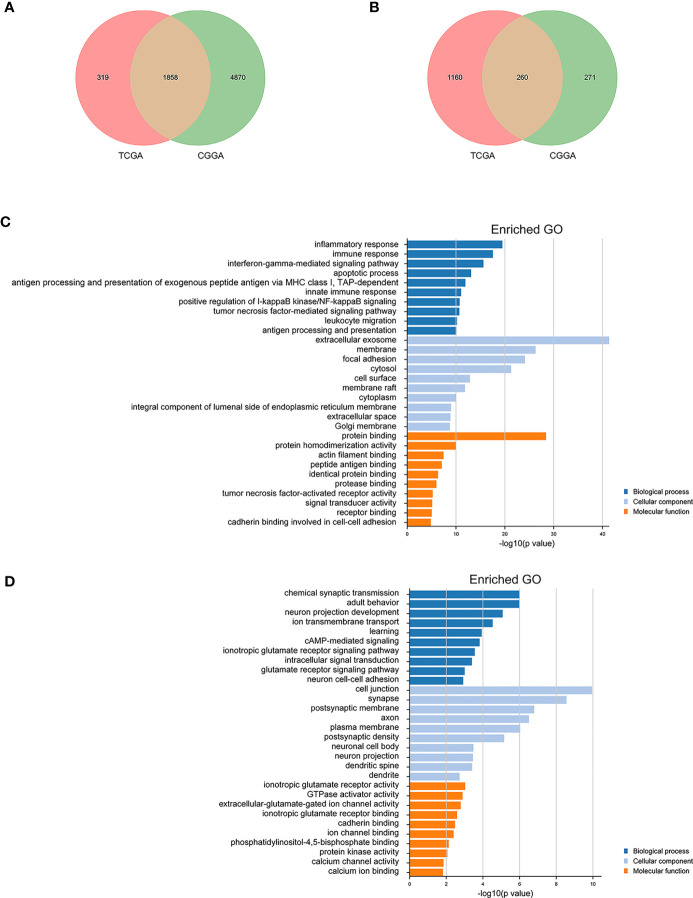
Gene ontology (GO) analysis of ARID5A-related genes in glioma based on CGGA and TCGA datasets. A total of 1858 copositive genes and 260 conegative genes were found in CGGA and TCGA **(A, B)**. GO analyses of the correlation genes **(C, D)**.

To further understand the role of ARID5A in gliomas, Pearson correlation analysis was used to select genes closely related to the expression of ARID5A (|Pearson *r*| > 0.3) in CGGA and TCGA glioma datasets. A total of 1858 genes positively correlated with ARID5A expression, while 260 genes negatively correlated with ARID5A expression in the intersection of CGGA and TCGA datasets (**Figures 6A, B**). We then conducted GO analysis for ARID5A expression-related genes using DAVID. The top ten GO terms ranked by *P* value are listed (**Figures 6C, D**). The GO terms of co-expression genes were divided into biological processes (BP), cellular component (CC) and molecular function (MF) groups. According to BP analysis, genes positively correlated with ARID5A expression were mainly enriched in inflammatory response, immune response, interferon-gamma-mediated signaling pathway, apoptotic process, antigen processing and presentation of exogenous peptide antigen *via* MHC class I, TAP-dependent; genes negatively correlated with ARID5A expression were mainly enriched in chemical synaptic transmission, adult behavior, neuron projection development, ion transmembrane transport, and learning. For CC, genes positively correlated with ARID5A expression were significantly enriched in extracellular exosome, membrane, focal adhesion, cytosol, and cell surface; genes negatively correlated with ARID5A expression were significantly enriched in cell junction, synapse, postsynaptic membrane, axon, and plasma membrane. According to MF analysis, genes positively correlated with ARID5A expression were mainly enriched in protein binding, protein homodimerization activity, actin filament binding, peptide antigen binding, and identical protein binding; genes negatively correlated with ARID5A expression were mainly enriched in ionotropic glutamate receptor activity, GTPase activator activity, extracellular-glutamate-gated ion channel activity, ionotropic glutamate receptor binding, and cadherin binding. Our results indicate that the expression of ARID5A is probably associated with immune functions in glioma.

### GSEA of ARID5A

**Figure 7:**
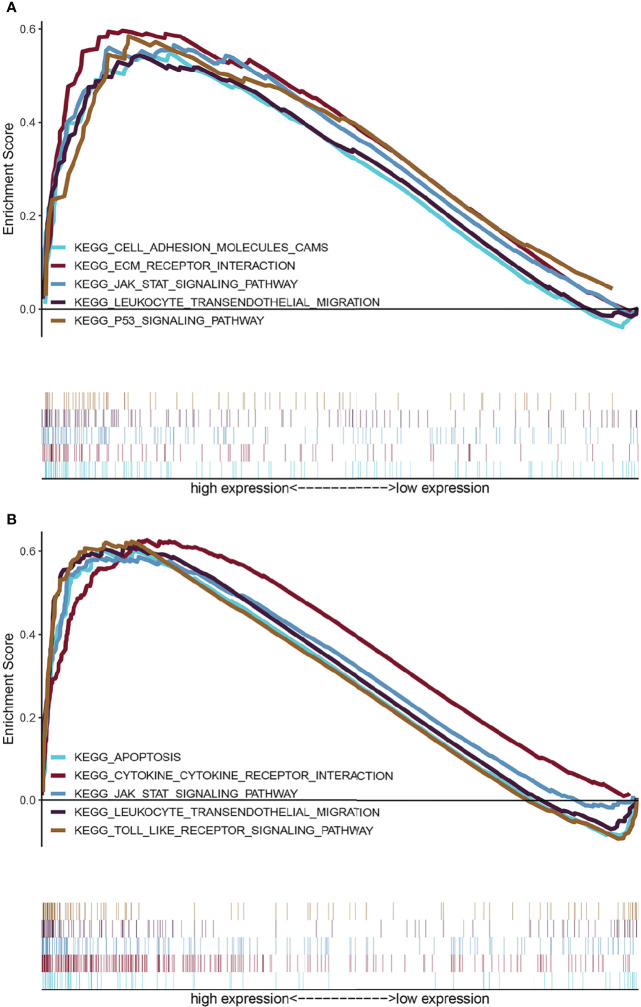
GSEA enrichment analysis of ARID5A using the CGGA **(A)** and TCGA **(B)** database.

To explore the influence of possible pathways of ARID5A in glioma, GSEA was performed on the datasets with high and low expression of ARID5A. Results showed that cell adhesion molecules (CAMs), the ECM receptor interaction, JAK-STAT signaling pathway, leukocyte transendothelial migration, and P53 signaling pathway were enriched in the high ARID5A expression group in CGGA datasets (**Figure 7A**). Apoptosis, cytokine-cytokine receptor interaction, JAK-STAT signaling pathway, leukocyte transendothelial migration and toll-like receptor signaling pathway were enriched in the high ARID5A expression group in TCGA datasets (**Figure 7B**). These results suggest that ARID5A may affect the occurrence, development and clinical prognosis of glioma by regulating the above-mentioned pathways of glioma patients, and a high level of ARID5A expression is associated with the regulation of cell apoptosis and immune response in glioma.

### ARID5A Expression Was Correlated With the Immune Infiltration Level and Cumulative Survival in Gliomas(LGG + GBM) From TIMER

**Figure 8:**
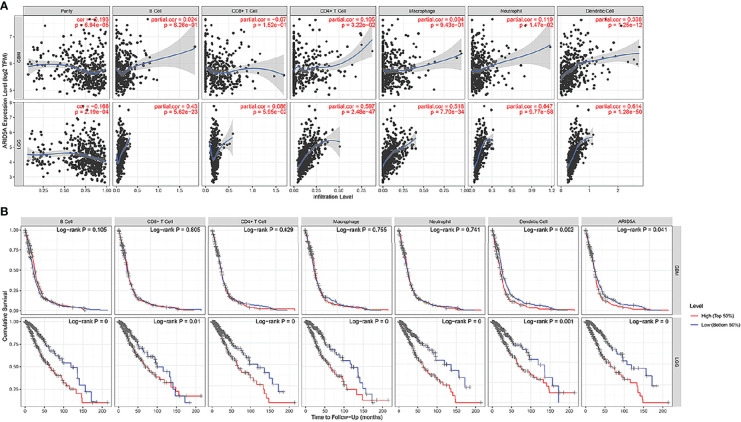
Correlation between ARID5A expression and infiltration levels of immune cells **(A)**.The correlation between survival and abundance of immune infiltration in GBM and LGG **(B)**. Blue shows a low degree of infiltration and red shows a high degree of infiltration.

The same type of tumor with different levels of immune infiltration had different clinical outcomes. We used the TIMER online analysis tool to study the correlation between ARID5A expression in glioma and the level of immune infiltration. In GBM, the results showed that there was no significant correlation between the expression level of ARID5A and infiltration of B cells, CD8+ T cells, and macrophages, and the expression level of ARID5A was positively correlated with CD4+ T cells (cor = 0.105, *P* = 3.22e-02), neutrophils (cor = 0.119, *P* = 1.47e-02), and dendritic cells (cor = 0.338, *P* = 1.25e-12) but negatively correlated with tumor purity (cor = -0.193, *P* = 6.94e-05)(**Figure 8A**). In LGG, the expression level of ARID5A was not significantly correlated with CD8+ T cell, positively correlated with B cells (cor = 0.43, *P* = 5.62e-23), CD4+ T cells (cor = 0.597, *P* = 2.48e-47), macrophages (cor = 0.518, *P* = 7.70e-34), neutrophils (cor = 0.647, *P* = 9.77e-58), and dendritic cells (cor = 0.614, *P* = 1.28e-50), and negatively correlated with tumor purity (cor = -0.168,*P* = 2.19e-04) (**Figure 8A**). Kaplan-Meier survival curves demonstrated that B cells, CD8+ T cells, CD4+ T cells, macrophages, neutrophils, dendritic cells, and the expression of ARID5A were associated with the survival of LGG patients, while dendritic cells and ARID5A expression were associated with the survival of GBM patients (**Figure 8B**). Multivariate Cox survival analysis showed that age, macrophage infiltration, and ARID5A expression could be independent prognostic factors for LGG (**Table 1**), while ARID5A was not an independent prognostic factor for GBM (**Table 2**). From the results above, we can conclude that ARID5A is correlated with immune infiltration in glioma.

**Table 1:** Multivariate analysis of the correlation of ARID5A expression and immune infiltrates in LGG.

	Coef	HR	95% CI_l	95% CI_u	P-value	Sig
Age	0.055	1.057	1.040	1.073	0.000	***
Gender(male)	0.180	1.198	0.817	1.756	0.356	
B cell	2.941	18.932	0.079	4556.961	0.293	
CD8 Tcell	5.003	148.813	0.156	141913.788	0.153	
CD4 Tcell	-0.933	0.393	0.000	947.353	0.814	
Macrophage	4.851	127.898	2.439	6707.702	0.016	*
Neutrophil	-4.674	0.009	0.000	30.498	0.258	
Dendritic cell	0.631	1.879	0.046	76.948	0.739	
ARID5A	0.310	1.364	1.058	1.758	0.017	*

LGG (n = 458), *P < 0.05; ***P < 0.001.

**Table 2:** Multivariate analysis of the correlation of ARID5A expression and immune infiltrates in GBM.

	Coef	HR	95% CI_l	95% CI_u	P-value	Sig
Age	0.032	1.032	1.025	1.040	0.000	***
Gender(male)	0.095	1.100	0.905	1.337	0.340	
B cell	-0.552	0.576	0.322	1.029	0.062	
CD8 Tcell	0.232	1.261	0.852	1.867	0.246	
CD4 Tcell	0.107	1.112	0.575	2.151	0.751	
Macrophage	0.208	1.232	0.657	2.311	0.516	
Neutrophil	-0.081	0.922	0.411	2.067	0.844	
Dendritic cell	0.369	1.446	1.099	1.901	0.008	**
ARID5A	0.163	1.177	0.959	1.444	0.118	

GBM (n = 402), **P < 0.01; ***P < 0.001.

## Discussion

Gliomas are central nervous system tumors that originate from the neuroectodermal layer and account for about 80% of malignant brain tumors (15). Currently, the main clinical treatment is a combination of operation and chemoradiotherapy (16). However, the recurrence rate of glioma is relatively high, the overall outcome remains poor, and the mortality of patients is relatively high (17, 18). In recent years, with the growing understanding of the molecular mechanisms related to the occurrence and development of glioma as well as the in-depth study of the central nervous system and the immune system, targeted therapy and immunotherapy for glioma have become the focus of clinical research, bringing new hope for the treatment of glioma (19–21). Therefore, it is of great clinical significance to search for biological indicators with important value for early diagnosis and prognosis of glioma.

ARID5A, a member of the ARID family, has seldom been studied in human tumors. Our findings show that ARID5A expression was upregulated in glioma compared with normal brain tissues by analyzing GEPIA and the expression of ARID5A in TCGA and the GTEx database. The survival analysis results showed that patients with high expression of ARID5A had worse overall survival in the TCGA and CGGA databases. In contrast, low expression of ARID5A in lung cancer is associated with poor prognosis (12). Furthermore, we investigated the correlation between the expression of ARID5A and the clinical and molecular characteristics of glioma patients. We found that the expression of ARID5A was significantly upregulated with increasing tumor grade and that high ARID5A expression was correlated with patient age, IDH wild-type gliomas, and 1p/19q non-codeletion gliomas. IDH mutation and 1p/19q co-deletion are early genetic events in the occurrence and progression of glioma (22, 23). Studies have shown that wild-type IDH, 1p/19q non-codeletion, and higher tumor grade were associated with poor prognosis in glioma (24, 25). These results indicate that ARID5A expression increases with the increase of tumor malignancy, suggesting that ARID5A can be used as an indicator of the tumor malignancy degree. ROC curve analysis proved that the ARID5A expression level has diagnostic value for gliomas. The GO analysis shows that upregulated ARID5A expression in glioma is highly correlated with inflammatory response, immune response, interferon-gamma-mediated signaling pathway, and apoptotic process, these biological functions may be potential molecular mechanisms affecting the growth and proliferation of cancer cells. Interferon-gamma-mediated signaling pathway has been identified as one of the vital signaling pathways in cancer (26). IFN‐γ is a proinflammatory cytokine produced by immune cells,including cytotoxic CD8+ T cells and CD4+ Th1 T cells (27). IFN‐γ exerts a dual effect on cancer immunology. On one hand, IFN-γ can inhibit the proliferation of tumor cells and induces MHC class I on tumor cells to enhance immunogenicity, and suppresses angiogenesis in tumors (26). On the other hand, continuous exposure to IFN‐γ can enhance tumor growth and facilitate the formation of tumor immunosuppressive microenvironment (28). Previous studies have indicated that ARID5A could enhance the production of IFN-γ by stabilizing T-bet mRNA (29, 30). Collectively, these findings indicated that ARID5A may be involved in the formation of tumor microenvironment through the interferon-gamma-mediated signaling pathway.

The results from GSEA showed that high expression of ARID5A was highly associated with the P53 signaling pathway, JAK-STAT signaling pathway, other tumor-related pathways, and some immune-related pathways. P53 is an important tumor suppressor gene which can regulate cell apoptosis, cell cycle and mediate DNA damage repair (31). Mutations in the P53 gene can lead to uncontrolled cell proliferation, which can lead to cancer (32). Accumulating evidence indicates that the occurrence and development of glioma are closely related to the expression level of P53 (33, 34). The JAK-STAT signaling pathway is one of the classic intracellular signal transduction pathways that regulates cell proliferation, differentiation, and apoptosis and also plays an important role in the tumorigenesis (35, 36). Studies have shown that the JAK-STAT signaling pathway plays an important role in the regulation of glioma cell survival, growth, and invasion and is one of the most important potential targets for gene therapy (37, 38). Previous studies have suggested that ARID5A can stabilize the mRNAs encoding the IL-6 and Stat3 genes (30), while IL-6 and/or STAT3 can modulate p53 expression in gene transcription and protein degradation (39, 40), suggesting that ARID5A may be associated with the P53 signaling pathway and JAK-STAT signaling pathway in tumor growth and development.

The tumor microenvironment (TME) plays an important role in the development of tumors and can be used as a biomarker for diagnosis and prognosis (41). The TME is composed of different cell types and extracellular matrix components, the cellular components mainly include tumor cells and a variety of immune cells, fibroblasts, vascular endothelial cells, and smooth-muscle cells. Non-cellular components of the TME mainly include extracellular matrix proteins, cytokines, and chemokines released by cells (42). TIICs mainly include tumor-associated macrophages, T lymphocytes, B lymphocytes, natural killer cells, dendritic cells, and polymorphonuclear leukocytes, which may play an important role in suppressing or promoting tumor growth and could affect prognosis of patients (43). Through the TIMER database analysis, we found that ARID5A expression was positively correlated with the infiltration of CD4+ T cells, neutrophils and dendritic cells in GBM, and the expression level of ARID5A was positively correlated with the infiltration of B cells, CD4+ T cells, macrophages, neutrophils, and dendritic cells in LGG. Tumor-infiltrating lymphocytes (TILs) are important components of the tumors immune microenvironment and play a vital role in the formation and progression of tumor, including CD8+ CTLs, CD4+ Th, CD4+/CD25+/FoxP3+ Treg (44). TILs have been related to a better outcome in various studies, but recent evidence have shown that TILs are associated with immunosuppression and immune evasion in the tumor microenvironment (44, 45). A recent study reported that cytolytic activity score (CYT) based on granzyme A and perforin expression were hallmarks of T cell infiltration and activation, and high CYT was associated with a more immunosuppressive tumor microenvironment and worse prognosis (46). In our study, we also found that the expression of ARID5A was positively correlated with TILs and negatively correlated with prognosis. These findings suggest that ARID5A may have some functional roles in TILs. However, the underlying mechanism by which ARID5A influences TILs in the tumor immune microenvironment needs further study. Neutrophils also have been shown to correlate with clinical outcomes in glioma. Elevated neutrophil count is associated with poor prognosis in diffuse glioma (47). In addition, Neutrophil infiltration correlated with glioma grade and worse survival among resistant patients (48, 49). Dendritic cells (DCs) are the most powerful antigen-presenting cell, which can recognize tumor antigens in a variety of tumors and activate a variety of immune cells, such as T lymphocytes, to produce anti-tumor immune responses (50). However, some studies have found that immature DCs can induce tumor immune tolerance and promote tumor growth (51). IL-6-mediated STAT3 activation inhibits the maturation and activation of DC (52), while ARID5A plays an important role in the IL‐6/STAT3 signaling pathway (53), suggesting that ARID5A may be related to the function of DC. These results indicate that ARID5A may be involved in immune response by affecting TIICs in tumor microenvironment. Furthermore, we also found that the expression of ARID5A was negatively associated with tumor purity in glioma, suggesting that ARID5A expression also is negatively associated with prognosis. Our findings were confirmed by previous studies showed that low tumor purity of tumor was correlated with poor prognosis (47, 54). However, there are some limitations in our study. Our study on ARID5A was based on database mining, cell lines and animal experiments have not been conducted to confirm the role of ARID5A in glioma. Therefore, the potential role of ARID5A in glioma and its underlying mechanism are required for further experimental study.

Some breakthroughs have been made in the treatment of glioma, but the prognosis of most patients is still poor. Therefore, the study of biomarkers which are associated with the occurrence and development of glioma is helpful to provide more evidence for the diagnosis and treatment of glioma. We found that ARID5A may be a prognostic and molecular target for glioma, which can help guide the treatment selection of glioma patients. Deep research of the expression and function of ARID5A in glioma can provide accurate diagnostic evidence and reliable prognostic information, and thus improve the clinical therapeutic level of glioma.

## Conclusion

In conclusion, our study discovered the biological function of ARID5A in glioma for the first time. The expression of ARID5A in glioma is associated with poor prognosis of glioma patients, and ARID5A expression in glioma is related to patient age, IDH mutation status, and 1p/19q co-deletion. In addition, ARID5A plays an important role in inflammation and immunity; we also found that ARID5A may play a vital role in regulating the immune microenvironment of glioma and thus affect the progression of glioma. Studying the function of ARID5A in glioma and its immune microenvironment will be helpful for us to better understand this cancer and could result in the identification of a new gene-targeted immunotherapy in glioma. Therefore, ARID5A can be used as a promising molecular predictor to evaluate the prognosis of glioma patients as well as a therapeutic target in the clinical detection of glioma.

## Data Availability Statement

Publicly available datasets were analyzed in this study. These data can be found here: CGGA, http://www.cgga.org.cn; TCGA, https://portal.gdc.cancer.gov/.

## Author Contributions

All authors contributed to the design and writing of the manuscript. All authors contributed to the article and approved the submitted version.

## Funding

This work was supported by Zhongnan Hospital of Wuhan University Science, Technology and Innovation Seed Fund (project no. znpy2018105).

## Conflict of Interest

The authors declare that the research was conducted in the absence of any commercial or financial relationships that could be construed as a potential conflict of interest.
